# *ZBED6* deletion activates *FNDC1*-mediated PI3K/AKT and MAPK/ERK1/2 signaling pathways to promote myogenesis in pigs

**DOI:** 10.1186/s40104-026-01504-w

**Published:** 2026-10-05

**Authors:** Xiao Zhang, Ruirui Pan, Miaomiao Qin, Jiakai Tang, Yifan Han, Chuxiong Zhang, Jiahao Chen, Haoyu Pei, Shan Jiang, Lijing Bai, Jiangwei Wu

**Affiliations:** 1 Key Laboratory of Animal Genetics, Breeding and Reproduction of Shaanxi Province, College of Animal Science and Technology, Northwest A&F University, Yangling, Shaanxi 712100 China; 2 Shenzhen Branch, Guangdong Laboratory for Lingnan Modern Agriculture, Genomics Institute at Shenzhen, Chinese Academy of Agricultural Sciences, Shenzhen, 518000 China

**Keywords:** FNDC1, MAPK/ERK1/2, Myogenesis, PI3K/AKT, ZBED6

## Abstract

**Background:**

Myogenesis is a tightly regulated biological process that is essential for skeletal muscle growth, regeneration, and livestock production. Although the transcription factor ZBED6 is a key regulator of skeletal muscle development, the downstream molecular mechanisms through which it controls myogenesis remain incompletely understood. Here, using ZBED6-deficient pigs and primary porcine skeletal muscle satellite cells, we sought to identify novel downstream targets of ZBED6 and elucidate the molecular mechanisms underlying ZBED6-mediated regulation of myogenesis.

**Results:**

By integrating transcriptomic, quantitative proteomic, and ChIP-seq datasets, we identified fibronectin type III domain-containing protein 1 (FNDC1), a muscle-derived secreted factor, as a previously unrecognized direct transcriptional target of ZBED6. ChIP-qPCR and dual-luciferase reporter assays showed that ZBED6 directly binds to the *FNDC1* promoter and represses its transcription. Functional analyses in primary porcine skeletal muscle satellite cells showed that *ZBED6* knockdown markedly promoted myoblast proliferation, differentiation, and myotube formation, whereas *FNDC1* knockdown partially attenuated these effects. Conversely, *FNDC1* overexpression partially rescued the impaired myogenesis caused by *ZBED6* overexpression. Mechanistically, *ZBED6* deficiency promoted porcine myoblast proliferation and differentiation, at least in part, through *FNDC1*-mediated activation of the PI3K/AKT and MAPK/ERK1/2 signaling pathways. Consistently, pharmacological inhibition of either pathway significantly attenuated the enhanced porcine myoblast proliferation and differentiation induced by *ZBED6* deficiency, supporting that activation of these signaling pathways contributes in part to the pro-myogenic effects mediated by the ZBED6-FNDC1 regulatory axis in porcine myoblasts.

**Conclusions:**

This study identifies *FNDC1* as a novel direct transcriptional target of ZBED6, supporting the ZBED6-FNDC1 axis as a regulatory mechanism contributing to porcine myoblast proliferation and differentiation. These findings expand the current understanding of the ZBED6 regulatory network and provide a cellular framework for future studies investigating skeletal muscle growth in vivo.

**Supplementary Information:**

The online version contains supplementary material available at https://doi.org/10.1186/s40104-026-01504-w.

## Introduction

Skeletal muscle is a crucial tissue that affects pork production performance and plays a significant role in animal productivity. Increasing the growth rate of skeletal muscle is of great significance for enhancing the meat production [1, 2]. Zinc finger BED domain-containing protein 6 (ZBED6) is a phylogenetically conserved transcription regulator in mammals, which is closely related to muscle growth and development [3, 4]. The discovery of ZBED6 arose from the establishment of inbred lines between European wild boars and Large White domestic pigs, which laid the foundation for the systematic study of quantitative trait loci (QTLs) for lean traits in domestic pigs [5, 6]. Studies in mice have shown that *Zbed6* deficiency leads to weight gain and enhanced skeletal muscle growth [7, 8]. Consistent with this, we previously showed that *ZBED6* deficiency significantly promotes skeletal muscle growth in pigs [9, 10]. These results suggest that ZBED6 acts as a negative regulator of skeletal muscle growth under physiological conditions. Functional studies on ZBED6-deficient pigs and *Zbed6*-knockdown C2C12 cells showed that ZBED6 plays an important regulatory role in development, wound healing, cell proliferation and growth [3, 8]. As a transcription factor, ZBED6 preferentially binds the consensus motif 5′-GCTCGC-3′, thereby regulating the transcription of numerous target genes involved in various physiological processes [11]. It regulates the expression of several genes, including insulin-like growth factor 2 (*IGF2*) [12, 13]. *IGF2* was the first identified target of ZBED6 during normal muscle growth in pigs and mice [3, 14]. Over 2,500 ZBED6 binding sites have been identified by chromatin immunoprecipitation sequencing (ChIP-seq) in C2C12 cells [3]. We previously showed that ZBED6 regulates multiple novel target genes in skeletal muscle, in addition to *IGF2* [10, 15]. We revealed that dedicator of cytokinesis 3 (*DOCK3*) is a direct target of ZBED6 in the circumstance of muscle atrophy in pigs [15]. We also uncovered several novel ZBED6 target genes, such as cyclin-dependent kinase inhibitor 1 A (*CDKN1A*), that control muscle development in placental mammals [10]. Several genes have been demonstrated to be targets of ZBED6, collectively contributing to the regulation of organ growth in pigs [10]. However, the functional role of ZBED6 in pigs with regard to other target genes remains to be fully elucidated. This study aims to further investigate the role of ZBED6, together with unexplored target genes, in regulating skeletal muscle growth in pigs.

Skeletal muscle is an important metabolic and locomotor organ whose growth and development are precisely regulated by multiple genes [1]. We recently identified FNDC1 as a novel myokine that promotes myogenesis and skeletal muscle growth under normal and injured conditions [16]. FNDC1 belongs to the fibronectin type III domain-containing protein family, which comprises 11 members. Our previous study showed that FNDC1 plays an important role in muscle growth and repair by promoting myoblast differentiation and myofiber formation. In addition, we found that recombinant FNDC1 effectively promotes muscle regeneration and alleviates the symptoms of muscular dystrophy, revealing the potential application of FNDC1 in the treatment of muscle diseases [16]. Studies have shown that the human aging process is strongly associated with a decline in skeletal muscle function, which is largely attributed to a decrease in the number of resident satellite cells (SCs) and their reduced activity [17, 18]. Mutations in the *FNDC1* gene were found to significantly exacerbate age-related muscle impairment in humans [19]. These findings emphasize the important role of *FNDC1* in the regulation of muscle growth and atrophy. However, the upstream regulatory mechanisms of* FNDC1* expression remain unclear, and the specific transcription factors involved in regulating *FNDC1* expression have not been investigated. This knowledge gap limits our comprehensive understanding of the regulatory network and mechanism of *FNDC1* action in skeletal muscle growth. Therefore, elucidating the upstream regulatory mechanism of *FNDC1* will not only help advance the molecular regulatory theory of muscle growth and development, but also provide new molecular targets for the treatment of muscle-related diseases. To identify the transcription factor of *FNDC1*, we screened its promoter region and revealed potential ZBED6 binding sites, which suggests that there may be a functional regulatory relationship between ZBED6 and *FNDC1*, and their interaction may play an important role in regulating skeletal muscle growth and development. By integrating multi-omics data from C2C12 cells with porcine skeletal muscle ChIP-seq data, we found that *FNDC1* is a potential direct target of ZBED6 in pigs, suggesting a potential relationship between ZBED6 and *FNDC1* in skeletal muscle growth and development.

Therefore, to investigate the role of the ZBED6-FNDC1 axis in the regulation of skeletal muscle growth, we used *ZBED6-*knockout pigs and their skeletal muscle satellite cells to study whether ZBED6 regulates skeletal muscle satellite cell proliferation and differentiation through *FNDC1*. We also performed RNA-sequencing and gene enrichment analysis to identify downstream signaling pathways involved in the ZBED6-FNDC1 axis. The PI3K/AKT and MAPK/ERK1/2 pathways were identified as downstream regulatory pathways involved in this axis. Our findings indicate that ZBED6 negatively regulates *FNDC1*, inhibiting FNDC1-mediated skeletal muscle growth. *ZBED6* deletion promotes myogenesis, at least in part, by enhancing *FNDC1* expression and subsequently activating the PI3K/AKT and MAPK/ERK1/2 signaling pathways. The study provides new insights into the roles of ZBED6 and *FNDC1* in skeletal muscle growth and reveals a novel regulatory interaction between the transcription factor ZBED6 and its target gene *FNDC1*. Further studies will help clarify the molecular mechanisms linking ZBED6 and *FNDC1* and provide candidate targets for increasing skeletal muscle growth rate and meat production in pigs.

## Materials and methods

### Animals and sample collection

Twelve healthy male Bama miniature pigs, including six wild-type (WT) pigs and six ZBED6-deficient pigs, were randomly selected from the F5 generation of our in-house breeding colony maintained at the Laboratory Animal Center of Chengdu Clonorgan Biotechnology Co., Ltd. Pigs were housed under environmentally controlled conditions at 25–28 °C and 60%–70% relative humidity and were provided ad libitum access to a standardized diet and clean water. The animals were genotyped before grouping, and WT littermates were used as controls. At 8 months of age, jugular blood was collected using sterile needles and vacuum blood collection tubes. After clotting at room temperature for 30 min, samples were centrifuged at 2,500 × *g* for 10 min at 4 °C [20], and the serum was stored at −80 °C. Following euthanasia, the longissimus dorsi (LD), gastrocnemius (GAS), soleus (SOL), extensor digitorum longus (EDL), and tibialis anterior (TA) muscles were collected from consistent anatomical sites. Visible connective and adipose tissues were removed, and the samples were immediately frozen in liquid nitrogen and stored at −80 °C until analysis. Body weights at the time of sample collection were 31 ± 0.8 kg for WT pigs and 35 ± 1.2 kg for ZBED6-deficient pigs.

### Isolation and culture of primary porcine skeletal muscle satellite cells

Primary porcine skeletal muscle satellite cells were isolated from the LD muscle of neonatal WT and *ZBED6* knockout piglets (< 1 week old, *n* = 3 piglets per genotype). Freshly collected muscle tissues were washed several times with cold phosphate-buffered saline (PBS) containing antibiotics, and visible connective tissue, adipose tissue, blood vessels, and nerve fibers were carefully removed. The muscle tissues were minced into small fragments and sequentially digested with 1.5 mg/mL protease, followed by collagenase type II, at 37 °C with intermittent gentle agitation. After enzymatic digestion, the cell suspension was mechanically dissociated by gentle pipetting, filtered sequentially through 100, 70, and 40 μm cell strainers, and centrifuged to collect mononuclear cells. The isolated cells were pre-plated in T75 culture flasks for 1 h at 37 °C in a humidified atmosphere containing 5% CO₂ to reduce fibroblast contamination. Non-adherent cells enriched for satellite cells were collected by centrifugation and cultured in pre-warmed proliferation medium at 37 °C with 5% CO₂ for subsequent experiments [21].

### Cell culture and proliferation assay

Primary porcine skeletal muscle satellite cells were seeded in 24-well plates at 2 × 10^4^ cells/well and cultured to approximately 70% confluence. For EdU analysis, cells were incubated with 10 μmol/L EdU for 2 h, fixed with 4% paraformaldehyde for 15 min, and processed using the EdU detection kit (MCE, HY-K1087) according to the manufacturer’s instructions. Nuclei were counterstained with DAPI. For each independent experiment, three randomly selected, non-overlapping fields per group were imaged at 200 × using an Olympus fluorescence microscope. EdU-positive and total DAPI-positive nuclei were quantified using ImageJ, and proliferation was expressed as the percentage of EdU-positive nuclei among total nuclei.

For the CCK-8 assay, cells were seeded in 96-well plates at 5 × 10^3^ cells/well and analyzed using the Cell Counting Kit-8 (Yeasen Biotechnology, 40203ES76) according to the manufacturer’s instructions. At the indicated time points, 10 μL of CCK-8 reagent was added to each well, followed by incubation at 37 °C for 2 h. Absorbance was measured at 450 nm using a microplate reader. Each independent experiment included five technical replicate wells per group, and blank wells without cells were used for background correction.

### siRNA and plasmid transfection

For transfection, primary porcine skeletal muscle satellite cells were seeded in 12-well plates at a density of 5 × 10^4^ cells/well and cultured for 24 h until they reached approximately 70% confluence. For gene knockdown, cells were transfected with siRNAs (50 nmol/L) targeting *FNDC1* or *ZBED6,* or with a negative control siRNA using Lipofectamine™ RNAiMAX Transfection Reagent (Invitrogen, 13778030). For gene overexpression, cells were transfected with pcDNA3.1-based expression plasmids encoding *FNDC1* or *ZBED6* (1 μg/well in a 12-well plate) or the corresponding empty vector control using Lipofectamine™ 3000 Transfection Reagent (Invitrogen, L3000001) according to the manufacturer’s instructions. At 24 h after transfection, the culture medium was replaced with differentiation medium consisting of DMEM supplemented with 2% horse serum and 1% penicillin/streptomycin. The differentiation medium was refreshed every 2 days. Cells and culture medium were collected for subsequent analyses. To evaluate knockdown or overexpression efficiency, cells were harvested at 24 h post-transfection for qPCR analysis and at 48 h post-transfection for Western blot analysis.

The siRNA sequences and qPCR primer sequences used in this study are listed in Table 1. The full-length coding sequences of porcine *FNDC1* and *ZBED6* were synthesized and cloned into the pcDNA3.1 expression vector, and all constructs were verified by Sanger sequencing.

**Table 1:** Primer and siRNA sequences used in this study

Primers	Sequence
sus-*ZBED6* For	5′-CTGTTATGTGCTTCCCCCAC-3′
sus-*ZBED6* Rev	5′-AGGGTAACTATCACACTAAGCTG-3′
sus-*FNDC1* For	5′-GGCATCCGAGTGGACAAAGA-3′
sus*-FNDC1 Rev*	5′-ACAATGGTTCGACCGTCTCC-3′
sus*-CCNE1 For*	5′-GGCCTGAGGTCTGAACTTGT-3′
sus-*CCNE1* Rev	5′-CGTGACACTTAGACACAGCA-3′
sus-*CDK4* For	5′-CCCATAGGCACCAACACCAA-3′
sus-*CDK4* Rev	5′-GGGAATTTGGTGGCAGGAGT-3′
sus-*CCND1* For	5′-CCCCCTCTGCGGAGACTTG-3′
sus-*CCND1* Rev	5′-TGGGCATTAGGGACTCTCGG-3′
sus-*PCNA* For	5′-GCAGATGTACCCCTTGTTGTAG-3′
sus-*PCNA* Rev	5′-TCACCGAAGCAGTTCTCAAAGA-3′
sus-*MYOD* For	5′-CCTAAAGCCCGAGGAACACT-3′
sus-*MYOD* Rev	5′-TAGTGGTCTTGCGTTTGCAC-3′
sus-*MYOG* For	5′-AACCCCACTTCTATGACGGG-3′
sus-*MYOG* Rev	5′-TTATCTTCCAGGGGCACTCG-3′
sus-*MYF5* For	5′-GAGTTCGGGGACGAGTTTGA-3′
sus-*MYF5* Rev	5′-TTTCCTCTTGCACGCTTTGC-3′
sus-*GAPDH* For	5′-CGGAGTGAACGGATTTGGC-3′
sus-*GAPDH* Rev	5′-ATGACAAGCTTCCCGTTCTC-3′
sus-*IGF2* For	5′-GTGCTGCTATGCTGCTTACCG-3′
sus-*IGF2* Rev	5′-CCGCAGACAAACTGGAGGG-3′
si-*ZBED6* For	5′-GGCAGUUCUUUGUGUAACATT-3′
si-*ZBED6* Rev	5′-UGUUACACAAAGAACUGCCTT-3′
si-*FNDC1* For	5′-ACUUUCAGGCCCAUUUUAGUG-3′
si-*FNDC1* Rev	5′-CUAAAAUGGGCCUGAAAGUCA-3′
si-NC For	5′-UUCUCCGAACGUGUCACGU-3′
si-NC Rev	5′-ACGUGACACGUUCGGAGAA-3′
*FNDC1* promoter-ChIP-qPCR-F	5′-AGATGCTGTGCCCAGATCG-3′
*FNDC1* promoter-ChIP-qPCR-R	5′-GTCCTGTAAGCACGCGAACA-3′
*GAPDH* ChIP-qPCR-F	5′-CATATCCTCCCGTTACATCC-3′
*GAPDH* ChIP-qPCR-R	5′-CCTGGTTCTGCCAATGTG-3′

### Pathway inhibitor treatment

Primary porcine skeletal muscle satellite cells were treated with 10 μmol/L LY294002, a PI3K inhibitor (MCE, HY-10108), or 1 μmol/L SCH772984, an ERK1/2 inhibitor (MCE, HY-50846). Both inhibitors were dissolved in DMSO, with the final DMSO concentration maintained below 0.1% in all groups. For proliferation assays, inhibitors were added 24 h before analysis and maintained until EdU labeling or CCK-8 measurement. For differentiation assays, inhibitors were added at the onset of differentiation and replenished during medium changes every 48 h until sample collection. Inhibition of the corresponding pathways was confirmed by immunoblotting for phosphorylated and total AKT and ERK1/2.

### Real-time quantitative PCR (qPCR)

Total RNA was extracted from primary porcine skeletal muscle satellite cells and skeletal muscle tissues using TRIzol Reagent (Invitrogen, 15596026) according to the manufacturer’s instructions. Genomic DNA was removed during reverse transcription using the genomic DNA removal components included in the reverse-transcription kit. RNA concentration and purity were assessed using a NanoDrop 2000 spectrophotometer, and samples with an A_260_/A_280_ ratio of 1.8–2.1 were used for cDNA synthesis. A total of 1 μg of RNA was reverse-transcribed using the HiScript III All-in-one RT SuperMix Perfect for qPCR (Vazyme, R333-01) in a final reaction volume of 20 μL.

qPCR was performed on a CFX96 Real-Time PCR Detection System (Bio-Rad) in 20 μL reactions containing 10 μL of ChamQ SYBR qPCR Master Mix (Vazyme, Q311-02), 1 μL of diluted cDNA, 0.6 μL of each primer, and 7.8 μL of nuclease-free water. The amplification conditions were as follows: initial denaturation at 95 °C for 3 min, followed by 38 cycles of denaturation at 95 °C for 15 s and annealing/extension at 60 °C for 30 s. Melting-curve analysis was performed from 65 to 95 °C to confirm the specificity of amplification. Relative gene expression was calculated using the 2^−ΔΔCt^ method, with *GAPDH* used as the reference gene.

### Immunoblotting

Cells and skeletal muscle tissues were lysed in RIPA lysis buffer supplemented with protease and phosphatase inhibitor cocktails. Protein concentrations were determined using a bicinchoninic acid (BCA) protein assay kit. Equal amounts of protein (30 μg/lane) were separated by 8%–12% SDS–PAGE and transferred onto polyvinylidene difluoride (PVDF) membranes. After blocking with 5% non-fat milk in Tris-buffered saline containing 0.1% Tween-20 (TBST) for 1 h at room temperature, the membranes were incubated with the primary antibodies listed in Table 2 overnight at 4 °C. The membranes were subsequently washed with TBST and incubated with the appropriate horseradish peroxidase (HRP)-conjugated secondary antibodies for 1 h at room temperature. Protein bands were visualized using an enhanced chemiluminescence substrate, imaged using a ChemiDoc XRS + imaging system (Bio-Rad, Hercules, CA, USA), and quantified using ImageJ. Target-protein levels were normalized to GAPDH or α-tubulin, as appropriate.

**Table 2:** Antibodies used in this study

Antibodies	Source	Identifier
ZBED6	ATLAS	HPA068807
FNDC1	Atagenix Laboratories	ATA29400
α-Tubulin	Proteintech	11224-1-AP
MYHC	Abcam	ab124205
MYOG	Merck	M5815
PCNA	Abmart	TB2757
GAPDH	Proteintech	60004-1-Ig
p-AKT Ser473	Proteintech	66444-1-Ig
AKT	Cell Signaling	#9272
p-mTOR	Cell Signaling	#5536
mTOR	Cell Signaling	#2983
p-ERK1/2	Diagbio	db14364
ERK1/2	Abmart	T40071
Goat anti-rabbit (HRP)	Abbkine	A21020
Goat anti-mouse (HRP)	Abbkine	A21010
Goat anti-rabbit (Alexa Fluor 546)	Invitrogen	A-11035
Goat anti-mouse (Alexa Fluor 546)	Invitrogen	A-11003
IgG	Abcam	ab171870
p38	Immunoway	YT3513
p-p38	Wanlei	WLP1576
JNK1/2/3	Immunoway	YT2440
p-JNK1/2/3	Immunoway	YP0157
Histone H3 antibody	Abcam	ab1791

### Determination of serum FNDC1 levels

Serum FNDC1 concentrations were measured using a porcine FNDC1 ELISA kit (Jingmei Biotechnology, JM-10466P1) according to the manufacturer’s instructions. Serum samples were analyzed without dilution in duplicate. Absorbance was measured at 450 nm using a microplate reader. FNDC1 concentrations were calculated from a four-parameter logistic standard curve and expressed as pg/mL.

### Luciferase assay

Primary porcine skeletal muscle satellite cells were isolated from wild-type and ZBED6-deficient pigs and cultured as described above. The *FNDC1* promoter region was amplified from WT pigs and cloned into the pGL3-Basic plasmid carrying firefly luciferase (Promega). Cells were seeded into 24-well plates in DMEM supplemented with 10% serum and then transfected with 1 µg of the pGL3-*FNDC1* vector or the empty pGL3 vector as the control. In addition, 50 ng of the PRL-TK vector (Renilla luciferase; Promega) was co-transfected as an internal reference. After 48 h, luciferase activity was detected using the Dual-Luciferase Reporter Assay Kit (Yeasen Biotechnology, 11402ES60) and a luminometer [15, 22]. Five independent biological replicates were performed.

### ChIP-qPCR

Chromatin immunoprecipitation followed by quantitative PCR (ChIP-qPCR) was performed using primary porcine skeletal muscle satellite cells with a ChIP Kit (Abcam, ab500) according to the manufacturer's instructions. Briefly, cells were cross-linked with 1% formaldehyde, and the reaction was quenched with glycine. Cross-linked chromatin was sonicated to generate DNA fragments of approximately 200–500 bp. An aliquot of the fragmented chromatin was retained as the input control for normalization, while the remaining chromatin was divided into separate aliquots and immunoprecipitated overnight at 4 °C with an anti-ZBED6 antibody (Atlas Antibodies, HPA068807), ChIP-grade normal rabbit IgG (Abcam, ab171870), or an anti-Histone H3 antibody (Abcam, ab1791). Normal rabbit IgG was used as the negative immunoprecipitation control to assess nonspecific binding, whereas the Histone H3 antibody served as a positive control to verify the efficiency of the ChIP procedure.

Following reversal of cross-links and DNA purification, enrichment of the predicted ZBED6-binding region within the *FNDC1* promoter was determined by quantitative PCR using the percent-input method. DNA immunoprecipitated with the Histone H3 antibody was analyzed using primers targeting the *GAPDH* promoter as a positive-control genomic region to confirm successful chromatin immunoprecipitation. Primer sequences for the *FNDC1* promoter and the positive-control region are provided in Table 1.

### Immunofluorescence

Immunofluorescence staining was performed on differentiation day 6 in WT and *ZBED6*-knockout primary porcine skeletal muscle satellite cells transfected with si-control or si-*FNDC1*, as well as WT and *ZBED6*-overexpressing primary porcine skeletal muscle satellite cells transfected with pcDNA3.1 or the *FNDC1* overexpression vector. Cells were fixed with 4% paraformaldehyde for 20 min, permeabilized on ice with 0.5% Triton X-100 for 10 min, and subsequently blocked with 5% bovine serum albumin in PBS for 2 h at room temperature. Samples were incubated with primary antibodies in blocking buffer overnight at 4 °C. After PBS washing, cells were incubated with the appropriate fluorophore-conjugated secondary antibodies for 2 h at room temperature, followed by DAPI staining for 5 min. Fluorescence images were captured using BioTek Gen5. The fusion index was calculated as the percentage of MYHC-positive myotubes containing at least three nuclei relative to the total number of MYHC-positive myotubes. Three randomly selected fields from each of three independent experiments were analyzed using ImageJ [23]. Details of the antibodies used are shown in Table 2.

### RNA sequencing, quantitative proteomics and ChIP-seq analysis

Previously generated RNA-seq and stable isotope labeling by amino acids in cell culture–mass spectrometry (SILAC–MS) quantitative proteomic data from *Zbed6*-knockout mouse C2C12 myoblasts were obtained from reference [13], and *Zbed6* ChIP-seq datasets generated in mouse C2C12 myoblasts were obtained from references [3, 7]. For these previously published datasets, the published differential expression results were used for downstream filtering. Differentially expressed transcripts and proteins were defined using an adjusted *P* value < 0.05 and an absolute log_2_ fold change (|log_2_FC|) > 1.

RNA-seq expression matrices from ZBED6-deficient pigs (RPKM for PRJNA663759) and *Fndc1*-knockdown C2C12 cells (FPKM for GSE217125) were downloaded from the corresponding public datasets and analyzed in R (v4.4.1). Differential expression analysis was performed using the limma package (v3.60.6) based on linear modeling and empirical Bayes moderation. *P* values were adjusted for multiple testing using the Benjamini–Hochberg procedure, and genes with an FDR < 0.05 and |log_2_FC| > 1 were considered differentially expressed.

Gene set enrichment analysis (GSEA) was performed using clusterProfiler (v4.12.6) with genes ranked according to their log_2_ fold changes. Gene annotation and identifier conversion were conducted using biomaRt (v2.60.1), and KEGG pathway enrichment analysis was performed using species-specific annotations. Results were visualized using enrichplot and ggplot2 (v4.0.1).

### Statistical analysis

Data are presented as mean ± standard error of the mean (SEM). Statistical comparisons between two groups were performed using two-tailed Student's *t*-tests. For comparisons among more than two groups in a single-factor experimental design, one-way ANOVA followed by Tukey's post hoc test was used. For experiments involving two independent factors, two-way ANOVA followed by Sidak's multiple comparisons test was used. For image-based quantification, measurements obtained from multiple microscopic fields within the same independent experiment were averaged, and the resulting mean was treated as one biological replicate for statistical analysis. Microscopic fields were not treated as independent replicates. Significance levels were defined as ^*^P < 0.05, ^**^P < 0.01, and ^***^P < 0.001.

## Results

### The newly identified myokine *FNDC1* is a direct transcriptional target negatively regulated by the skeletal muscle regulator ZBED6

**Fig. 1:**
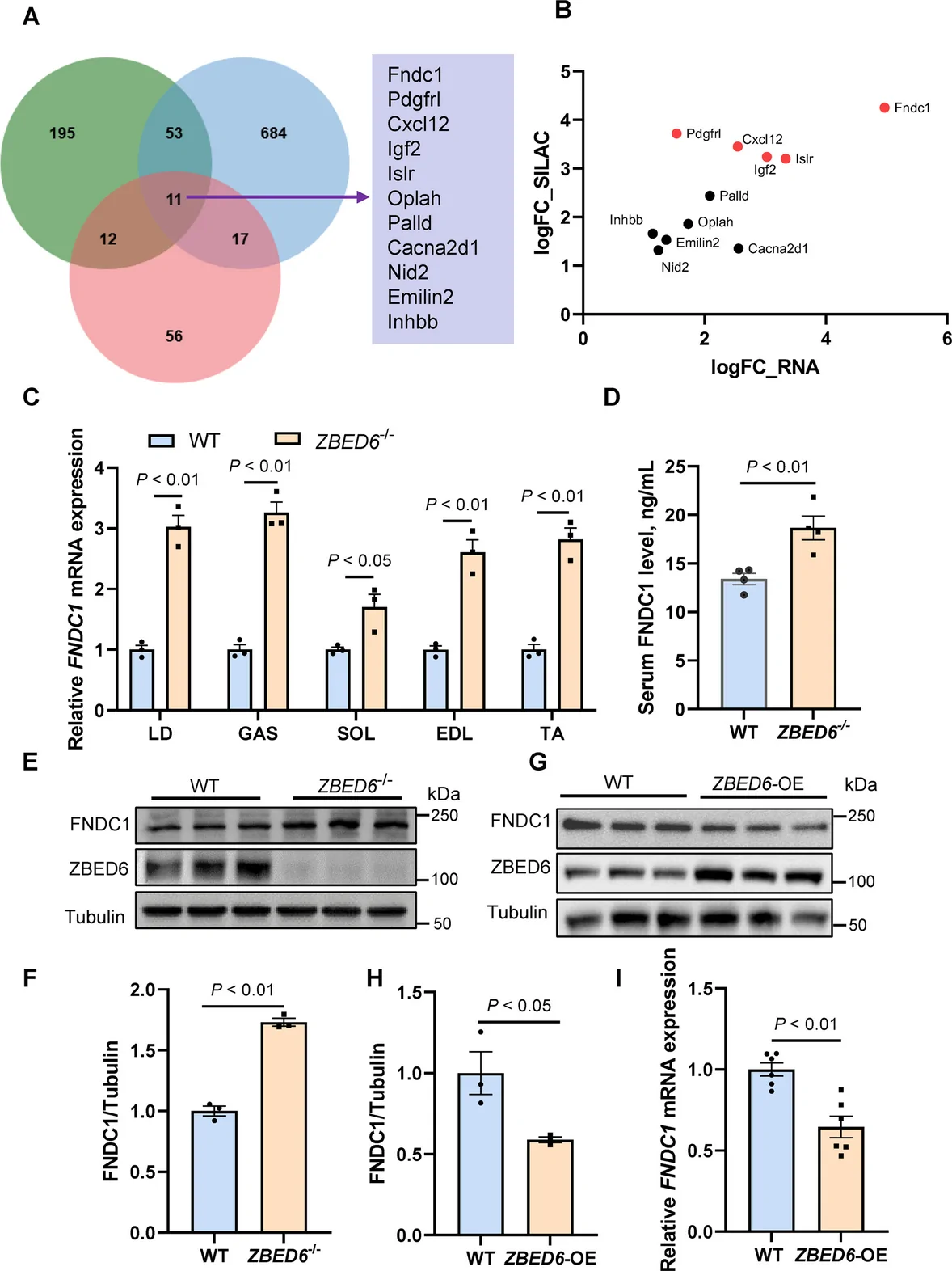
Regulation of *FNDC1* expression by ZBED6. **A** Multi-omics data from C2C12 cells were analyzed and integrated with porcine skeletal muscle ChIP-seq data to screen 11 candidate targets potentially regulated by ZBED6. **B** Scatter plot showing the log_2_ fold change of candidate genes at the RNA and SILAC protein levels. Red dots indicate the five selected candidate target genes used for further validation. **C** Relative mRNA expression levels of *FNDC1* in different muscle tissues of WT and *ZBED6* KO pigs, *n* = 3 pigs/genotype. LD: Longissimus dorsi muscle; GAS: Gastrocnemius; SOL: Soleus; EDL: Extensor digitorum longus; TA: Tibialis anterior. **D** FNDC1 levels in serum of WT and ZBED6 KO pigs, *n* = 4 pigs/genotype. **E** and **F** Protein expression levels and statistical analysis of FNDC1 in WT and ZBED6*-*deficient pigs, *n* = 3 pigs/genotype. **G **and** H** Protein expression levels of FNDC1 after overexpression of *ZBED6* in primary porcine skeletal muscle satellite cells and statistical analysis, *n* = 3 independent cell-culture experiments. **I** Relative mRNA expression levels of *FNDC1* in primary porcine skeletal muscle satellite cells after overexpression of *ZBED6*, *n* = 6 independent cell-culture experiments. Data are expressed as mean ± SEM. In (C), (D), (F), (H), and (I), the *P* values were determined by two-tailed Student's *t*-tests

To identify novel downstream effectors of ZBED6 involved in myogenesis, we integrated previously published RNA-seq and quantitative proteomics data [13] with ChIP-seq from C2C12 cells [7] and identified 11 candidate genes (*Fndc1*, *Pdgfrl*, *Cxcl12*, *Igf2*, *Islr*, *Oplah*, *Palld*, *Cacna2d1*, *Nid2*, *Emilin2*, *Inhbb*) potentially regulated by ZBED6 (Fig. 1A). These 11 candidate genes were ranked according to the magnitude of their differential expression (log_2_FC) in both the RNA-seq and SILAC datasets, and 5 highest-ranked genes, *Igf2*, *Fndc1*, *Cxcl12*, *Islr*, and *Pdgfrl*, were selected for further analysis (Fig. 1B). Given the conserved ZBED6-responsive sequences across mammals, promoter analysis of their porcine orthologs showed that the porcine *FNDC1* promoter contained the most abundant putative ZBED6-responsive motifs with a GCTCGN-like core sequence, designated as ZBED6-binding sites (ZBSs). Further RT-qPCR analysis of five skeletal muscle tissues, along with serum measurements, revealed that *FNDC1* mRNA levels were significantly increased in *ZBED6* knockout pigs (Fig. 1C and D). Western blot validation showed that FNDC1 levels were dramatically upregulated in skeletal muscles of ZBED6-deficient pigs (Fig. 1E and F). Additionally, in porcine muscle satellite cells, the expression of *FNDC1* was significantly downregulated by *ZBED6* overexpression (Fig. 1G–I), indicating that *FNDC1* is a candidate target of ZBED6 in pigs.

**Fig. 2:**
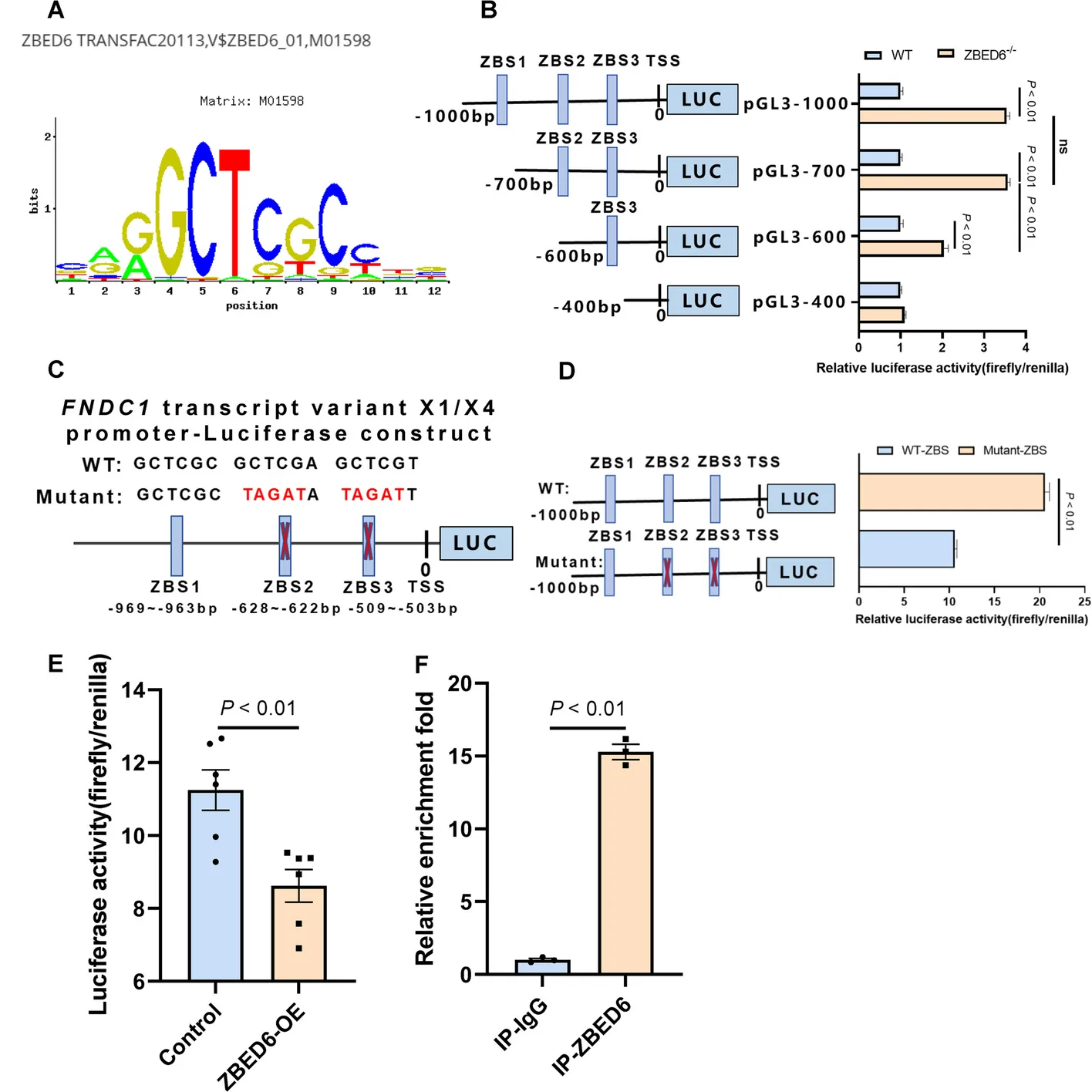
The novel myokine *FNDC1* is a direct negative target of the skeletal muscle regulator ZBED6. **A** Contra v3 analysis generated sequence identification of the ZBED6 binding site (ZBS) in the *FNDC1* promoter region. **B** Schematic illustration of serial *FNDC1* promoter deletion constructs containing different predicted ZBED6-binding sites (ZBS1–ZBS3) and corresponding dual-luciferase reporter activities. ZBS1, ZBS2, and ZBS3 were located at −969 to −963 bp, − 628 to −622 bp, and −509 to −503 bp relative to the TSS. **C** Schematic representation of the wild-type (WT) and mutant *FNDC1* promoter luciferase reporter constructs. **D** Dual luciferase activity of wild-type FNDC1-ZBS luciferase reporter vector (WT-ZBS) and mutant FNDC1-ZBS luciferase reporter vector (mut-ZBS) in pig primary muscle satellite cells. **E** Luciferase assay displaying the relative luciferase activity of the FNDC1-ZBS-LUC reporter in *ZBED6* overexpressing (*ZBED6* OE) and control (pcDNA3.1) cells, *n* = 6 independent cell-culture experiments. **F** ChIP analysis of ZBED6 on FNDC1-ZBS promoter region was performed in primary porcine skeletal muscle satellite cells, *n* = 3 independent cell-culture experiments. Data are expressed as mean ± SEM. In (B), (D), (E) and (F), the *P* values were determined by two-tailed Student's *t*-tests

To determine whether ZBED6 regulates *FNDC1* expression in pigs, we analyzed and characterized the ZBED6-binding sites within the *FNDC1* promoter using the ConTra v3 database (http://bioit2.irc.ugent.be/contra/v3/) (Fig. 2A). Sequence analysis identified three putative *ZBED6*-responsive sites containing a GCTCGN-like core sequence within the porcine *FNDC1* promoter, which were designated as ZBS1–ZBS3 (Fig. 2B). These three putative sites were located at −969 to −963 bp (ZBS1), − 628 to −622 bp (ZBS2), and −509 to −503 bp (ZBS3) relative to the transcription start site (TSS), respectively. ZBS1 was located upstream of the −700 bp promoter region, whereas ZBS2 and ZBS3 were located within the −700 to −400 bp region analyzed in the promoter deletion assays. To determine whether ZBED6 regulates *FNDC1* transcription, a series of *FNDC1* promoter fragments of different lengths (−1,000 to 0, − 700 to 0, − 600 to 0, and −400 to 0 bp) were cloned into the pGL3-Basic luciferase reporter vector and transfected into primary porcine skeletal muscle satellite cells. Luciferase reporter assays showed that the activity of the full-length (−1,000 bp) *FNDC1* promoter was approximately 3.5-fold higher in ZBED6-deficient cells than in wild-type cells (Fig. 2B). Promoter deletion analysis further revealed that this difference was maintained in the −700 bp construct but was markedly reduced in the −600 bp construct and almost abolished in the −400 bp construct, indicating that the major ZBED6-responsive region is located between −700 and −400 bp of the *FNDC1* promoter (Fig. 2B). To further validate this observation, mutation of the putative ZBED6-responsive sites in the *FNDC1* promoter (ZBS2 and ZBS3) increased reporter activity by approximately twofold relative to the wild-type promoter (Fig. 2C and D). Overexpression of *ZBED6* in wild-type pig satellite cells significantly reduced reporter activity compared with the control vector (Fig. 2E). In addition, ChIP-qPCR demonstrated that ZBED6 directly binds to the *FNDC1* promoter in primary porcine skeletal muscle satellite cells (Fig. 2F). These results suggest that *FNDC1* is a novel direct transcriptional target gene regulated by ZBED6.

### *ZBED6* knockdown promotes primary porcine skeletal muscle satellite cell proliferation by up-regulating *FNDC1*

**Fig. 3:**
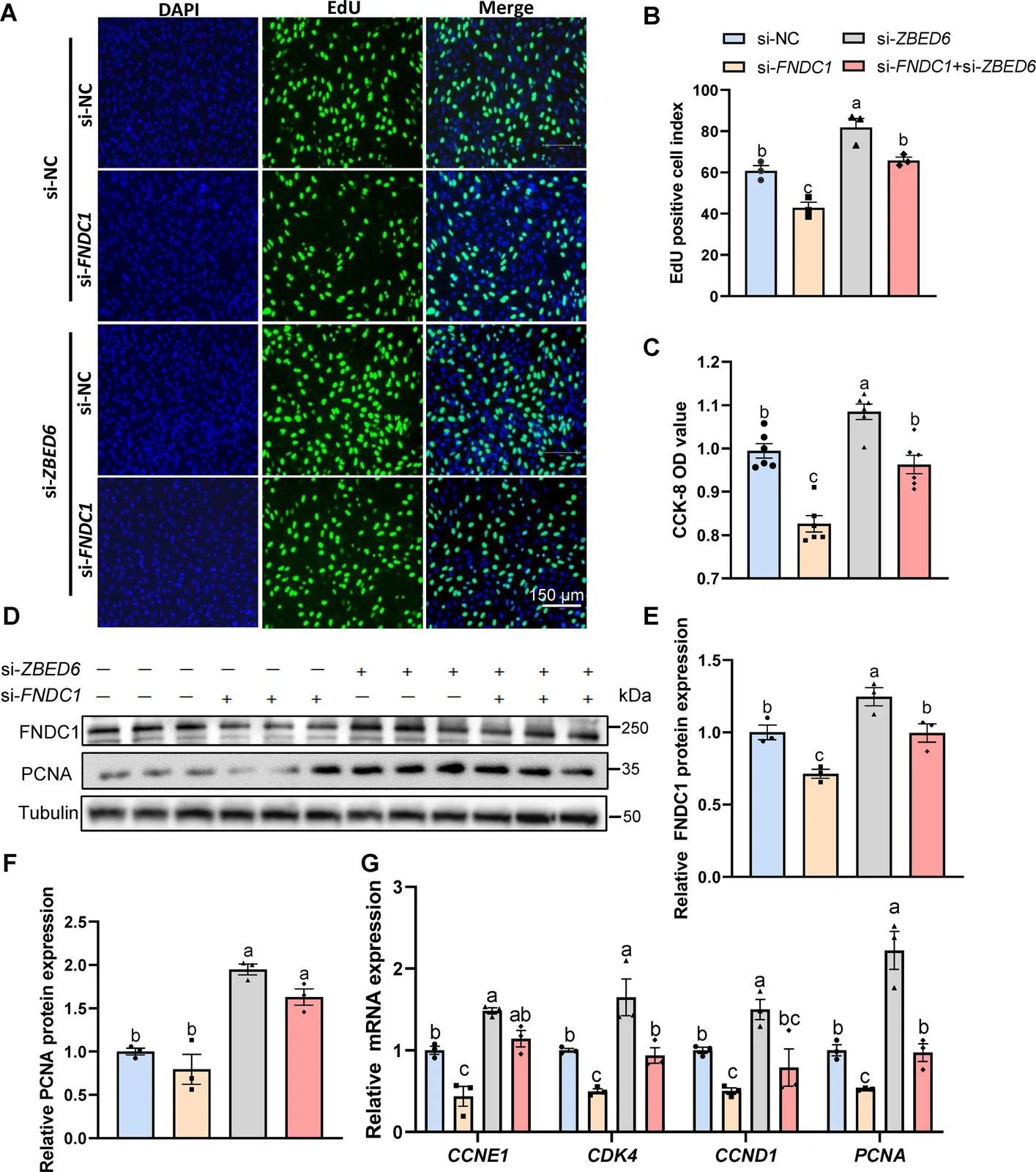
*ZBED6* deletion promotes primary porcine skeletal muscle satellite cell proliferation by up-regulating *FNDC1*. **A **and** B** Representative immunofluorescence staining for EdU (**A**) and percentage of EdU positive cells (**B**) in ZBED6-deficient primary porcine skeletal muscle satellite cells with control (si-NC) and *FNDC1* KD (si-*FNDC1*), *n* = 3 independent cell-culture experiments. **C** CCK-8 OD value in ZBED6-deficient primary porcine skeletal muscle satellite cells with control (si-*NC*) and *FNDC1* KD (si-*FNDC1*), *n* = 6 independent cell-culture experiments. **D–F** Protein expression levels and statistical analysis (right) of FNDC1 and PCNA, *n* = 3 independent cell-culture experiments. **G** Relative mRNA expression levels of *CCNE1*, *CDK4*, *CCND1* and *PCNA*, *n* = 3 independent cell-culture experiments. Data are expressed as mean ± SEM. In (B), (C), (E), (F), and (G), the *P* values were determined by two-way ANOVA followed by Sidak's multiple comparisons test, different letters (a, b, and c) between bars indicate *P* < 0.05

To investigate the role of FNDC1 in *ZBED6* deficiency-induced muscle growth, we used siRNA to knock down endogenous *ZBED6* in porcine muscle satellite cells (Fig. S1A). EdU staining revealed that *ZBED6* knockdown significantly increased the number of EdU-positive cells compared with controls (Fig. 3A and B). Interestingly, *FNDC1* knockdown significantly attenuated the increased cell proliferation induced by *ZBED6* knockdown (Fig. 3A and B, Fig. S1B). In addition, we further verified cell proliferation using the CCK-8 assay. Consistent with the EdU results, *ZBED6* knockdown significantly increased cell viability. Moreover, the enhanced cell viability induced by *ZBED6* knockdown was significantly reduced upon *FNDC1* knockdown (Fig. 3C). Previous studies identified *IGF2* as a direct and functionally important target of ZBED6 in myogenesis. To define the regulatory relationship among *ZBED6*, *IGF2*, and *FNDC1* in myoblast proliferation, we knocked down *IGF2* and *FNDC1* individually or simultaneously in ZBED6-deficient muscle satellite cells. Compared with the control group, *IGF2* knockdown did not affect *FNDC1* expression (Fig. S2A) and cell proliferation (Fig. S2C and D), whereas *FNDC1* knockdown did not alter *IGF2* expression (Fig. S2B) but significantly reduced cell proliferation (Fig. S2C and D). Collectively, these results suggest that *FNDC1* partially mediates the enhanced myoblast proliferation induced by *ZBED6* knockdown.

In *ZBED6*-knockdown porcine muscle satellite cells, protein levels of proliferating cell nuclear antigen (PCNA), a marker of cell proliferation, and FNDC1 were significantly upregulated (Fig. 3D–F). However, *FNDC1* silencing partially attenuated the increase in PCNA expression induced by *ZBED6* knockdown (Fig. 3D–F). Consistent with these findings, qPCR analysis showed that mRNA levels of *PCNA* and other positive proliferation regulators, including Cyclin E1 (*CCNE1*), Cyclin Dependent Kinase 4 (*CDK4*) and Cyclin D1 (*CCND1*), were significantly upregulated in *ZBED6-*knockdown porcine muscle satellite cells whereas knockdown of *FNDC1* markedly attenuated this upregulation (Fig. 3G). These findings indicate that *FNDC1* partially mediates the enhanced proliferation induced by *ZBED6* knockdown, consistent with the observed changes in proliferation-associated gene expression.

### *ZBED6* overexpression inhibits proliferation of primary porcine skeletal muscle satellite cells through down-regulation of *FNDC1*

**Fig. 4:**
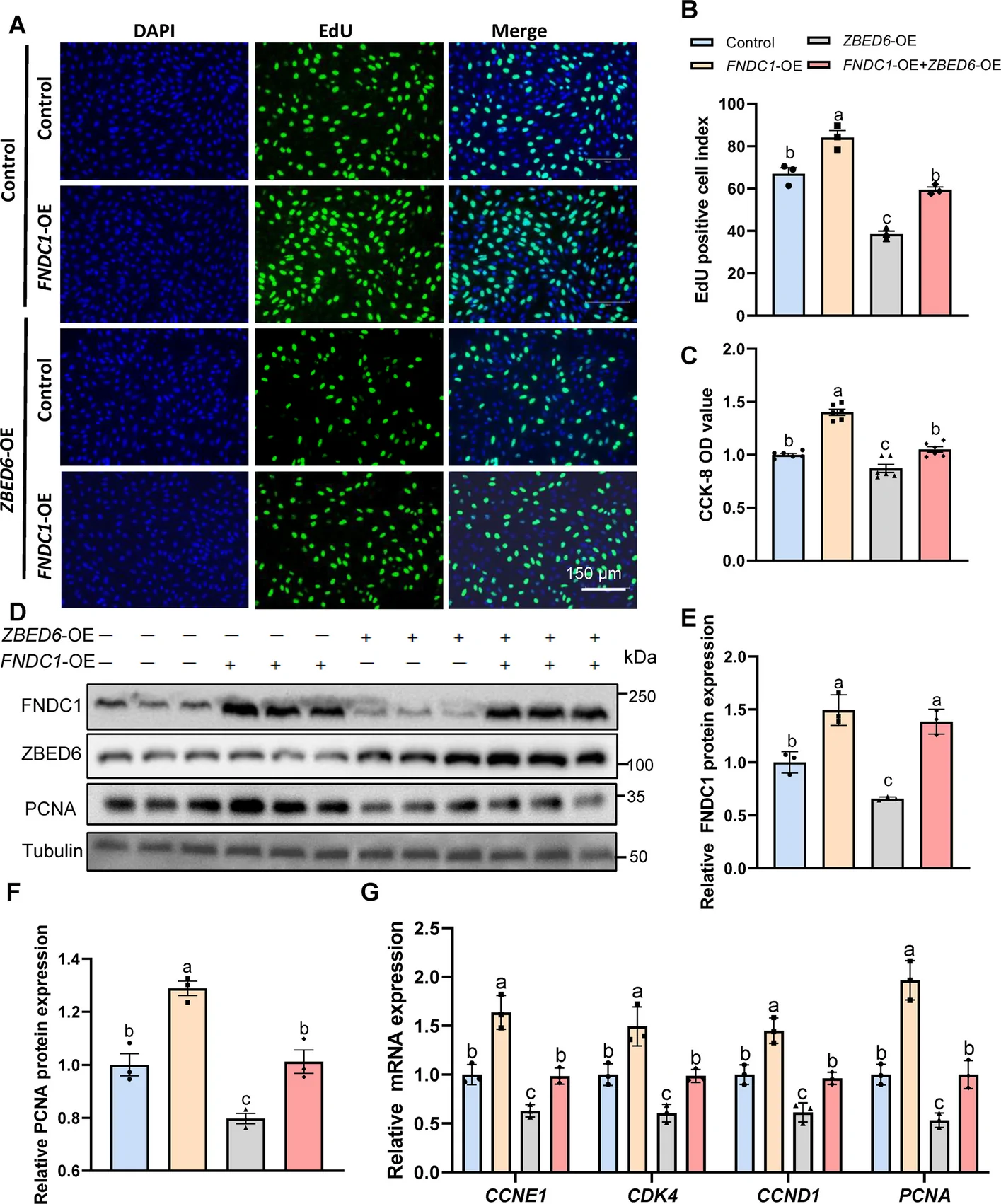
*ZBED6* overexpression inhibits proliferation of primary porcine skeletal muscle satellite cells through down-regulation of *FNDC1*. **A **and** B** Representative immunofluorescence staining for EdU (**A**) and percentage of EdU positive cells (**B**) in *ZBED6* overexpression primary porcine skeletal muscle satellite cells with control (NC) and *FNDC1* overexpression, *n* = 3 independent cell-culture experiments. **C** CCK-8 OD value in *ZBED6* overexpression primary porcine skeletal muscle satellite cells with control (NC) and *FNDC1* overexpression, *n* = 6 independent cell-culture experiments. **D **and** E** Protein expression levels (Left) and statistical analysis (Right) of FNDC1, *n* = 3 independent cell-culture experiments. **F** Protein expression levels and statistical analysis of PCNA in D, *n* = 3 independent cell-culture experiments. **G** Relative mRNA expression levels of *CCNE1*, *CDK4*, *CCND1* and *PCNA*, *n* = 3 independent cell-culture experiments. Data are expressed as mean ± SEM. In (B), (C), (E), (F), and (G), the *P* values were determined by two-way ANOVA followed by Sidak's multiple comparisons test, different letters (a, b and c) between bars indicate *P* < 0.05

We further investigated the effects of overexpression of *ZBED6* on the proliferation of primary porcine skeletal muscle satellite cells. EdU staining showed that in porcine muscle satellite cells, *ZBED6* overexpression significantly decreased the number of EdU-positive cells compared with the control (Fig. 4A and B, Fig. S3A), suggesting that *ZBED6* overexpression inhibited cell proliferation. In contrast, *FNDC1* overexpression significantly increased the number of EdU-positive cells in *ZBED6-*overexpressing porcine muscle satellite cells (Fig. 4A and B, Fig. S3B). In addition, the CCK-8 assay further confirmed this finding: overexpression of *ZBED6* significantly inhibited cell viability, whereas overexpression of *FNDC1* markedly ameliorated the ZBED6-induced decrease in viability (Fig. 4C).

Consistent with the knockdown results, *ZBED6* overexpression significantly decreased PCNA and FNDC1 protein levels (Fig. 4D–F), whereas *FNDC1* overexpression rescued the decrease in *PCNA* expression in *ZBED6*-overexpressing cells (Fig. 4D–F). Likewise, the mRNA levels of key positive regulators of proliferation were significantly downregulated by *ZBED6* overexpression; this downregulation was substantially alleviated when *FNDC1* was co-overexpressed with *ZBED6* (Fig. 4G). Together, these findings indicate that *ZBED6* inhibits satellite cell proliferation at least in part by suppressing *FNDC1* expression.

Collectively, the consistent results from *ZBED6* knockdown and overexpression experiments demonstrate that *ZBED6* regulates cell proliferation, at least in part, by suppressing *FNDC1*.

### *ZBED6* depletion promotes myoblast differentiation and myotube formation, at least in part, through upregulation of *FNDC1*

**Fig. 5:**
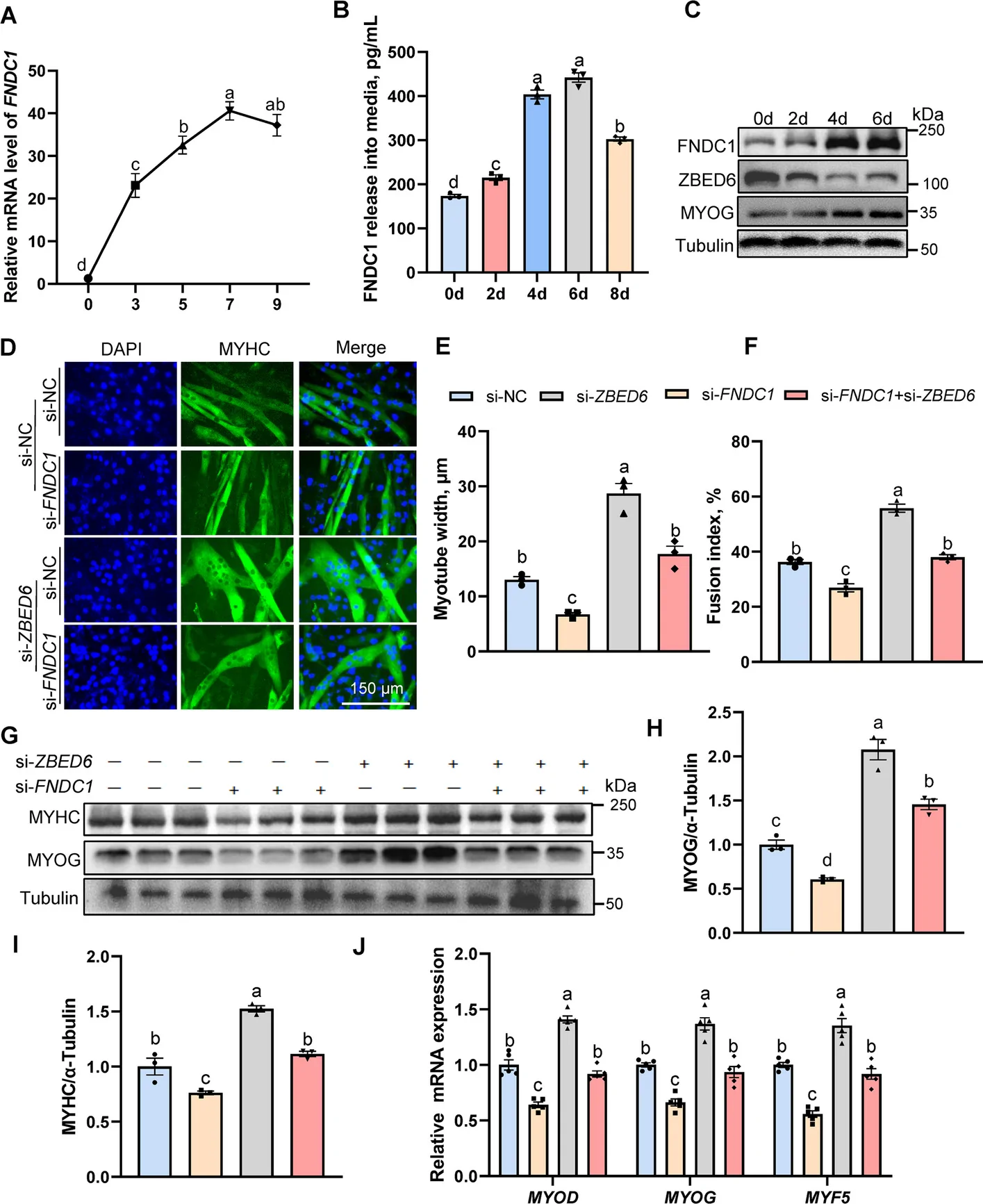
*ZBED6* depletion promotes myoblast differentiation and myotube formation via upregulation of *FNDC1*. **A** Relative mRNA expression of *FNDC1* during differentiation, *n* = 3 independent cell-culture experiments. **B** Secretion levels of FNDC1 in culture medium during myoblast differentiation, *n* = 3 independent cell-culture experiments. **C** Protein expression levels of FNDC1, MYOG and ZBED6 (0 d, 2 d, 4 d, 6 d). **D–F** Representative immunofluorescence staining for MYHC (**D**) and myotube width (**E**) and fusion index (**F**) in ZBED6-deficient primary porcine skeletal muscle satellite cells with control (si-NC) and *FNDC1* KD (si-*FNDC1*), *n* = 3 independent cell-culture experiments. **G–I** Protein expression levels and statistical analysis of MYHC and MYOG, *n* = 3 independent cell-culture experiments. **J** Relative mRNA expression levels of *MYOD*, *MYOG* and *MYF5*, *n* = 5 independent cell-culture experiments. Data are expressed as mean ± SEM. In (A) and (B), the *P* values were determined by one-way ANOVA with Tukey's post hoc test, different letters (a, b, c and d) between bars indicate *P* < 0.05. In (E), (F), (H), (I), and (J), the *P* values were determined by two-way ANOVA followed by Sidak's multiple comparisons test, different letters (a, b and c) between bars indicate *P* < 0.05

To investigate the regulatory role of ZBED6 in myoblast differentiation and its relationship with FNDC1, we examined the level of secreted FNDC1 in the culture medium and intracellular *FNDC1* mRNA and protein levels during myoblast differentiation. *FNDC1* mRNA levels increased about 20-fold on the third day of differentiation and continued to increase with the differentiation process (Fig. 5A). Consistently, FNDC1 levels in the culture medium rose to approximately 1.24-fold on day 2 of differentiation and further reached nearly 2.6-fold by day 6 (Fig. 5B). In line with this, the protein levels of FNDC1 were also significantly upregulated during differentiation (Fig. 5C). Together, these data suggest that *FNDC1* is significantly activated during myoblast differentiation and plays an important role in differentiation regulation. During myoblast differentiation, the protein levels of ZBED6 showed a gradual reduction (Fig. 5C). Earlier work identified *IGF2* as a direct and functionally important target of ZBED6 during myogenesis. To define the regulatory relationships among *ZBED6, IGF2,* and *FNDC1* in myoblast differentiation, we performed individual or combined knockdown of *IGF2* and *FNDC1* in ZBED6-deficient muscle satellite cells. *IGF2* knockdown reduced differentiation by approximately 62% (Fig. S4A and B), whereas *FNDC1* knockdown resulted in a 33% reduction compared with controls (Fig. S4A and B). Strikingly, simultaneous knockdown of both genes led to an approximately 88% decrease in differentiation (Fig. S4A and B). Collectively, these results suggest that, in the absence of *ZBED6*, myoblast differentiation is regulated through two parallel and functionally additive pathways mediated by *IGF2* and *FNDC1*.

Immunofluorescence analysis revealed that knockdown of *ZBED6* in porcine muscle satellite cells resulted in a significant increase in myoblast differentiation, accompanied by increased myotube diameter and fusion index (Fig. 5D–F). However, when we knocked down *FNDC1* in addition to *ZBED6*, the pro-myogenic effect induced by *ZBED6* knockdown was greatly reduced (Fig. 5D–F). These results suggest that *FNDC1* partially mediates the pro-myogenic effect induced by *ZBED6* knockdown.

Western blot analysis showed that MYOG and MYHC, two markers of myogenic differentiation, were significantly upregulated in *ZBED6*-knockdown porcine muscle satellite cells (Fig. 5G–I), whereas simultaneous knockdown of *ZBED6* and *FNDC1* impaired this upregulation (Fig. 5G–I). qPCR analysis of *MYOD*, *MYOG* and *MYF5* in porcine muscle satellite cells showed that *ZBED6* knockdown significantly upregulated these positive differentiation regulators, whereas simultaneous knockdown of both *ZBED6* and *FNDC1* impaired their upregulation in *ZBED6* knockdown cells (Fig. 5J).

### *ZBED6* overexpression inhibits myoblast differentiation and myotube formation through down-regulation of *FNDC1*

**Fig. 6:**
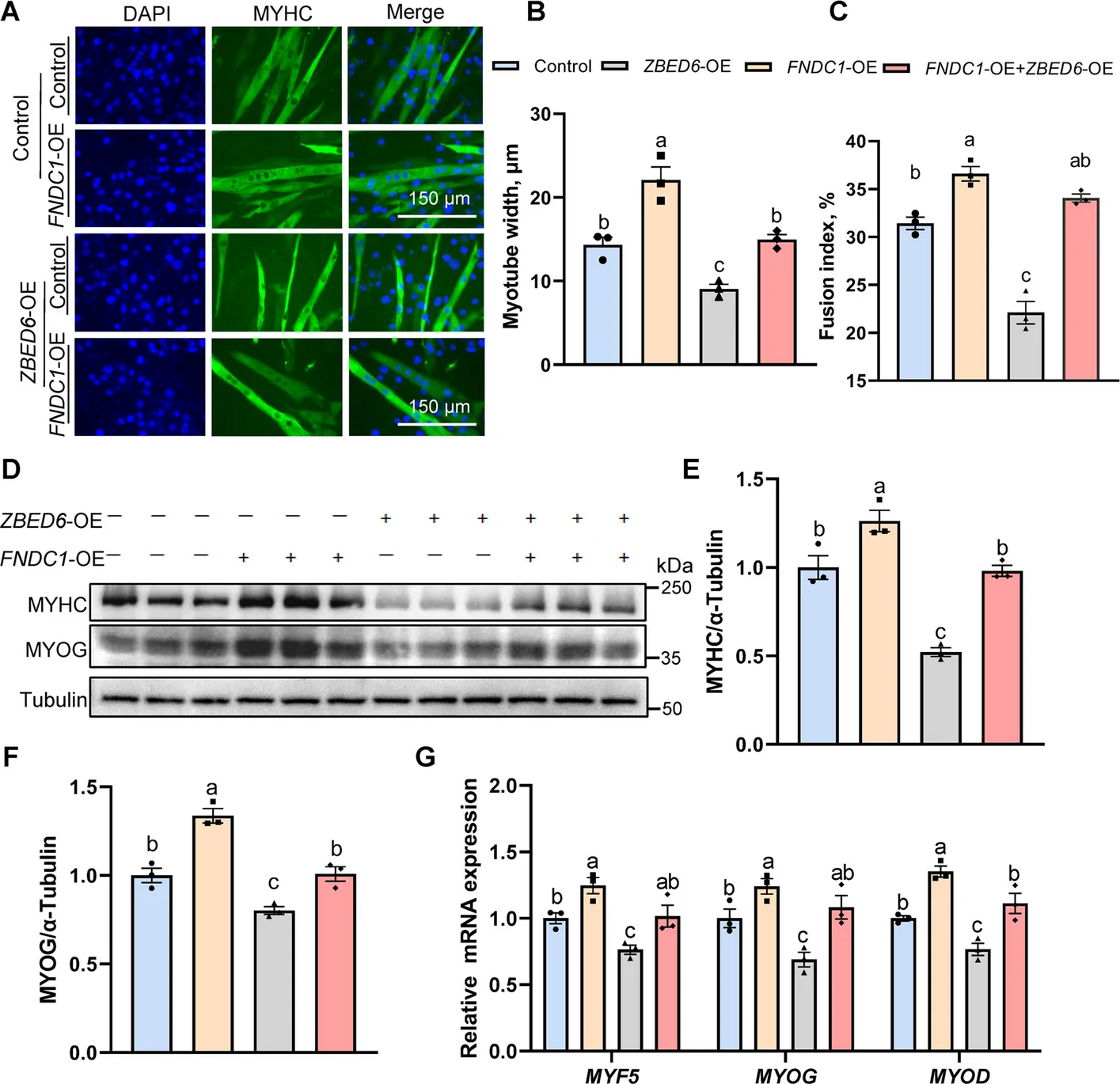
*ZBED6* overexpression inhibits myoblast differentiation and myotube formation through down-regulation of *FNDC1*. **A–C** Representative immunofluorescence staining for MYHC (**A**) and myotube width (**B**) and fusion index (**C**) in *ZBED6-*overexpressing primary porcine skeletal muscle satellite cells with control (NC) and *FNDC1* overexpression, *n* = 3 independent cell-culture experiments. **D–F** Protein expression levels and statistical analysis of MYHC and MYOG, *n* = 3 independent cell-culture experiments. **G** Relative mRNA expression levels of *MYF5*, *MYOG* and *MYOD*, *n* = 3 independent cell-culture experiments. Data are expressed as mean ± SEM. In (B), (C), (E), (F), and (G), the *P* values were determined by two-way ANOVA followed by Sidak's multiple comparisons test, different letters (a, b and c) between bars indicate *P* < 0.05

We further investigated the effects of *ZBED6* and *FNDC1* overexpression on the differentiation of primary porcine skeletal muscle satellite cells. Immunofluorescence analysis showed that overexpression of *ZBED6* in porcine muscle satellite cells significantly inhibited the differentiation of myoblasts, as evidenced by a significant decrease in the myotube diameter and fusion index (Fig. 6A–C). However, the inhibitory effect of *ZBED6* overexpression on myogenesis was significantly attenuated when *FNDC1* was co-overexpressed with *ZBED6* (Fig. 6A–C). These results suggest that *FNDC1* contributes to the inhibitory effects of *ZBED6* on myogenesis.

Western blot analysis showed that *ZBED6* overexpression in pig muscle satellite cells significantly decreased the protein levels of the muscle differentiation markers MYOG and MYHC (Fig. 6D–F). This inhibitory effect was markedly attenuated by co-overexpression of *FNDC1* (Fig. 6D–F). Consistently, qPCR analysis showed that *ZBED6* overexpression significantly downregulated the mRNA expression of *MYOD, MYOG,* and *MYF5*. In contrast, co-overexpression of *FNDC1* partially rescued the inhibitory effects of *ZBED6* on these myogenic genes (Fig. 6G). Together, these results indicate that *FNDC1* partially mediates the inhibitory effect of *ZBED6* on myoblast differentiation and myotube formation.

### *ZBED6* deletion activates FNDC1-mediated PI3K/AKT and MAPK/ERK1/2 signaling pathways to promote myogenesis

**Fig. 7:**
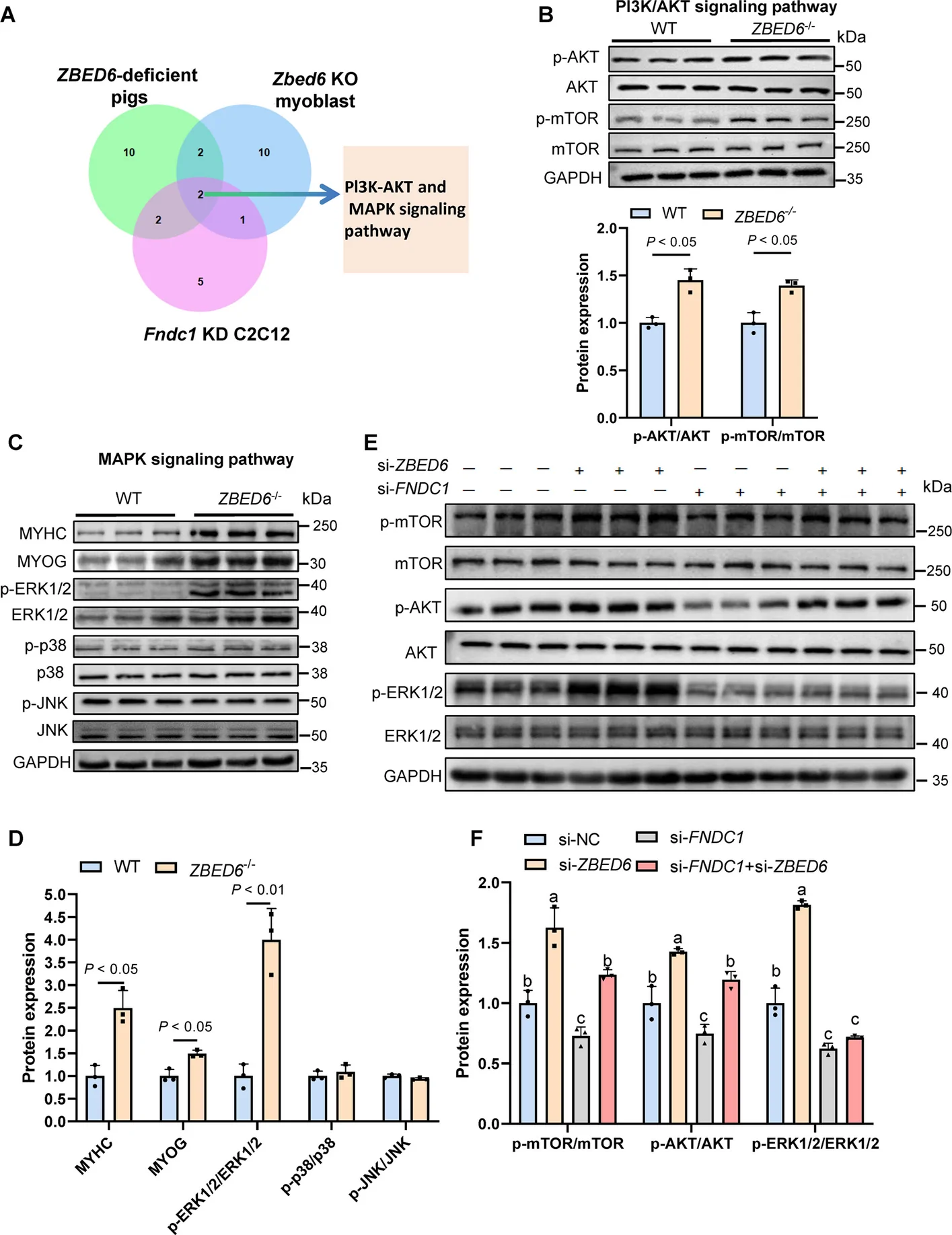
*ZBED6* deletion activates *FNDC1*-mediated PI3K/AKT and MAPK/ERK1/2 signaling pathways. **A** RNA-seq data from ZBED6-deficient pigs, *ZBED6* knockout myoblasts and myotubes, and *FNDC1* knockdown C2C12 cells were comprehensively analyzed to screen downstream signaling pathways. **B** Protein expression levels and statistical analysis of p-AKT, AKT, p-mTOR and mTOR in WT and *ZBED6*^*−/−*^ pigs, *n* = 3 pigs/genotype. **C **and** D** Protein expression levels and statistical analysis, *n* = 3 pigs/genotype. **E **and** F** Protein expression levels and statistical analysis of p-mTOR, mTOR, p-AKT, AKT, p-ERK1/2 and ERK1/2, *n* = 3 independent cell-culture experiments. Data are expressed as mean ± SEM. In (B), and (D), the *P* values were determined by two-tailed Student's *t*-tests. In (F), the *P* values were determined by two-way ANOVA followed by Sidak's multiple comparisons test, different letters (a, b and c) between bars indicate *P* < 0.05

To investigate the molecular mechanisms underlying the ZBED6-FNDC1 interaction during myogenesis, we analyzed RNA-seq datasets from ZBED6-deficient pigs (PRJNA663759), *Zbed6* knockout myoblasts [13], and *Fndc1*-knockdown C2C12 cells (GSE217125). Pathway enrichment analysis focused on pathways that were upregulated upon *ZBED6* deficiency (Fig. S5A, B and E) and downregulated upon *FNDC1* knockdown (Fig. S5C and D). We found that the PI3K/AKT and MAPK/ERK1/2 signaling pathways were consistently activated in ZBED6-deficient porcine muscle tissues and myoblasts, but suppressed in *FNDC1*-knockdown cells. These results suggest that the PI3K/AKT and MAPK/ERK1/2 pathways are downstream signaling pathways mediated by the *ZBED6-FNDC1* axis in promoting myoblast differentiation and myotube formation (Fig. 7A). To further validate these findings, we assessed the activation of PI3K/AKT and MAPK signaling pathways in skeletal muscle tissues from wild-type and *ZBED6* knockout pigs. Western blot analysis showed that the p-AKT/AKT and p-ERK1/2/ERK1/2 ratios were significantly higher in *ZBED6* knockout pigs than in wild-type controls, whereas p38 and JNK phosphorylation remained unchanged (Fig. 7B–D). These results indicate a specific association between ZBED6 and the AKT and ERK1/2 pathways.

To further determine whether *ZBED6* deficiency-induced activation of AKT, mTOR, and ERK1/2 signaling was dependent on FNDC1, we first showed that the ratios of p-AKT/AKT, p-mTOR/mTOR and p-ERK1/2/ERK1/2 were significantly increased (Fig. 7E and F), and that PCNA (Fig. 3F), MYOG and MYHC (Fig. 5G–I) were also significantly upregulated in *ZBED6*-knockdown porcine muscle satellite cells. However, when *ZBED6* and *FNDC1* were simultaneously knocked down in porcine muscle satellite cells, the increases in the ratios of p-AKT/AKT, p-mTOR/mTOR and p-ERK1/2/ERK1/2 (Fig. 7E and F), as well as the upregulation of PCNA (Fig. 3F), MYOG and MYHC (Fig. 5G–I), were impaired. These results suggest that the enhanced myogenesis induced by *ZBED6* knockdown in vitro is attributable, at least in part, to FNDC1-dependent activation PI3K/AKT and MAPK/ERK1/2 signaling pathways.

**Fig. 8:**
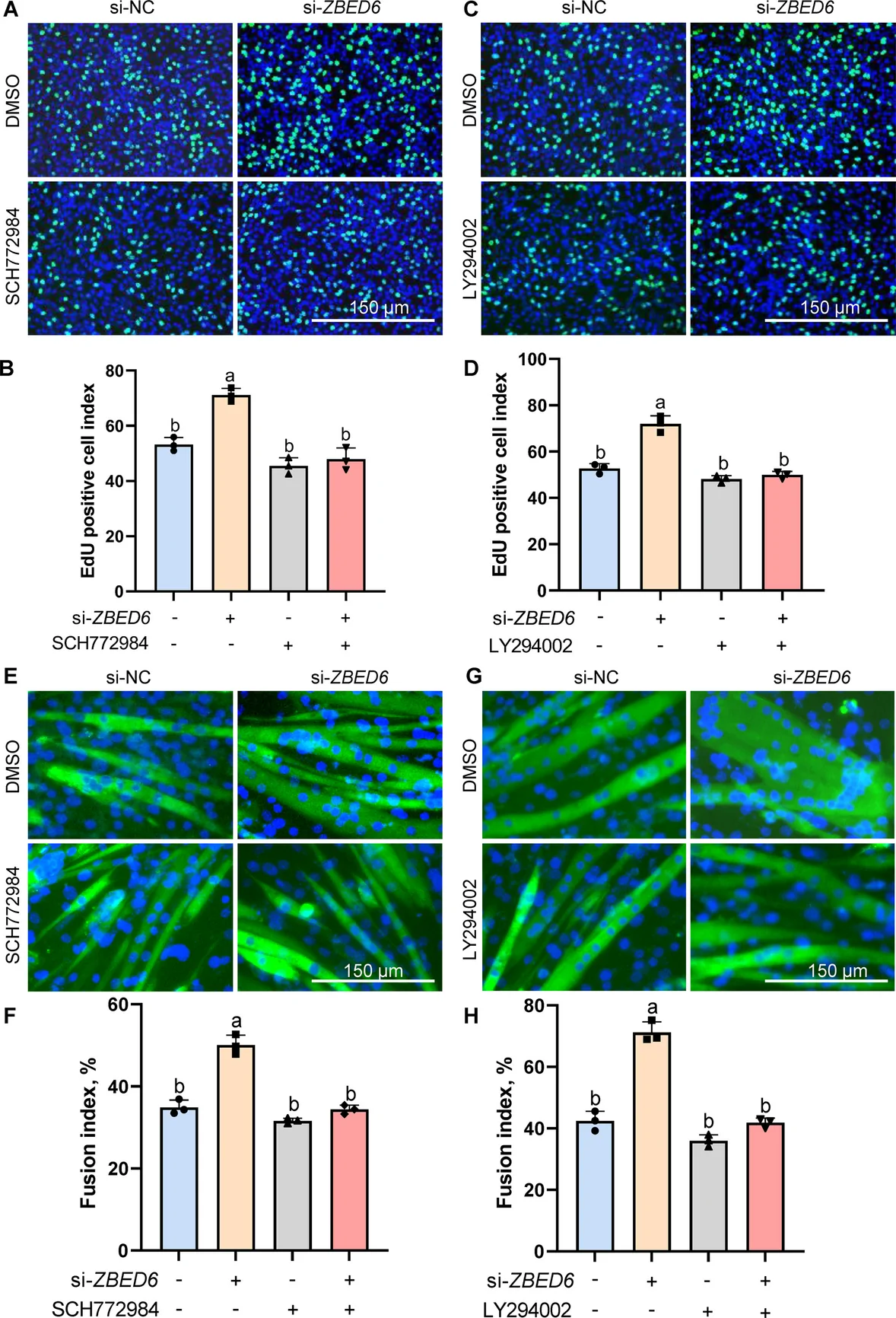
Inhibition of PI3K/AKT and MAPK/ERK1/2 pathways reduces the effects of the *ZBED6-FNDC1* axis on myogenesis. **A** and **B** representative immunofluorescence staining for EdU (**A**) and percentage of EdU positive cells (**B**) in *ZBED6* knockdown primary porcine skeletal muscle satellite cells treated with SCH772984,* n* = 3 independent cell-culture experiments. **C** and **D**. representative immunofluorescence staining for EdU (**C**) and percentage of EdU positive cells (**D**) in *ZBED6* knockdown primary porcine skeletal muscle satellite cells treated with LY294002, *n* = 3 independent cell-culture experiments. **E** and **F** representative immunofluorescence staining for MYHC (**E**) and fusion index (**F**) in *ZBED6* knockdown primary porcine skeletal muscle satellite cells treated with SCH772984, *n* = 3 independent cell-culture experiments. **G** and **H** representative immunofluorescence staining for MYHC (**G**) and fusion index (**H**) in *ZBED6* knockdown primary porcine skeletal muscle satellite cells treated with LY294002, *n* = 3 independent cell-culture experiments. Data are represented as mean ± SEM. In (B), (D), (F), and (H), the *P* values were determined by two-way ANOVA followed by Sidak's multiple comparisons test, different letters (a and b) between bars indicate *P* < 0.05

The results of the EdU assay and immunofluorescence staining indicated that the PI3K/AKT and MAPK/ERK1/2 signaling pathways contribute to ZBED6-regulated myogenesis (Fig. 8A–H). Treatment with either LY294002, a PI3K pathway inhibitor, or SCH772984, an ERK1/2 inhibitor, significantly attenuated the enhanced cell proliferation and myogenic differentiation induced by ZBED6 knockdown in vitro (Fig. 8A–H), supporting the involvement of PI3K/AKT/mTOR and MAPK/ERK1/2 signaling in si-*ZBED6*-regulated myogenesis.

## Discussion

Skeletal muscle, as the largest tissue in mammals, plays a crucial role in maintaining body functions [24]. Skeletal muscle satellite cells, as the main stem cells for skeletal muscle regeneration, directly determine the developmental process, damage repair and regeneration potential of skeletal muscle through their proliferative activity and directed differentiation ability [25, 26]. In this study, we revealed for the first time the important role of the regulatory axis between the transcription factor *ZBED6* and the novel myogenic factor *FNDC1* (*ZBED6-FNDC1* axis) in porcine myogenesis. Based on our previous studies, we have elucidated that ZBED6 plays a key role in skeletal muscle development by regulating downstream target genes (e.g., *CDKN1A*), and screened several potential target genes through multi-omics analysis [10, 15]. In this study, we identified the myokine FNDC1 as a potential direct target of ZBED6 by integrative analysis. This finding further revealed the molecular regulatory mechanism of ZBED6 in skeletal muscle. Further studies revealed that FNDC1, as a positive regulator of muscle growth, significantly promotes myogenesis [16]. By integrating bioinformatics analysis and experimental validation, we revealed that ZBED6 represses the transcriptional activity of *FNDC1* by directly binding to its promoter region, thus establishing the *ZBED6-FNDC1* axis as one important signaling pathway for regulating myogenesis. The discovery of this regulatory axis is of dual scientific significance: on the one hand, identifying *FNDC1* as a new downstream target of ZBED6 expands the regulatory network of ZBED6 and provides a new molecular basis for further analysis of its multifunctionality; on the other hand, placing FNDC1 within the downstream regulatory network of ZBED6 for the first time reveals a new regulatory hierarchy of FNDC1 in skeletal muscle growth and deepens our understanding of its biological role. Our study not only expands the functional network of ZBED6, but also provides a novel theoretical framework for the functional study of FNDC1, which lays an important molecular foundation for future muscle growth-related studies and applications.

Here we systematically characterized the regulatory relationship between the transcription factor ZBED6 and the new myogenic factor FNDC1, as well as the functional mechanism of this relationship in the proliferation and differentiation of primary porcine skeletal muscle satellite cells. We demonstrated that ZBED6 repressed the transcriptional activity of *FNDC1* by directly binding to its promoter region, thus establishing a regulatory network of the *ZBED6-FNDC1* axis in myogenesis. Further functional studies showed that *ZBED6* knockdown significantly promoted the proliferation and differentiation of primary porcine skeletal muscle satellite cells, whereas *FNDC1* knockdown exerted an opposite inhibitory effect. Notably, the si-*ZBED6*-mediated pro-proliferative and pro-differentiation effects were significantly attenuated when both *ZBED6* and *FNDC1* were knocked down, confirming that FNDC1 is an important downstream effector in the ZBED6-regulated proliferation and differentiation of skeletal muscle satellite cells. Previous studies have shown that *Zbed6* knockdown promotes cell proliferation but has no significant effect on *Igf2* expression, suggesting that these proliferative effects may be mediated by other target genes regulated by ZBED6 [3]. In the present study, we found that ZBED6 affected the proliferation efficiency of primary porcine skeletal muscle satellite cells, at least in part, through FNDC1, revealing a unique role of the *ZBED6-FNDC1* axis in porcine myogenesis. Notably, compared with our recent study[16], the present study suggests that the function of FNDC1 exhibited species specificity. We showed that in murine-derived muscle cells, FNDC1 treatment had no significant effect on cell proliferation [16], whereas in the present study, FNDC1 was found to significantly promote the proliferation of primary porcine skeletal muscle satellite cells. This inter-species functional difference may stem from the heterogeneity of the regulatory network, providing a new direction for future cross-species comparative studies. Importantly, our knockdown experiments indicate that FNDC1 only partially mediates the effects of *ZBED6* deficiency. Together with our *IGF2*-knockdown results, these findings support a model in which *FNDC1* and *IGF2* function as parallel downstream effectors of ZBED6, each contributing to distinct aspects of the myogenic phenotype.

By integrating multi-dimensional evidence from evolutionary biology and molecular genetics, we systematically elucidated the cross-species application potential of the ZBED6 regulatory pathway and its generalized scientific value in the study of muscle growth. ZBED6 is unique to placental mammals and is highly conserved among species with a near 100% amino acid identity across 26 placental mammals [3]. This high degree of sequence conservation implied that it may play a critical and conserved regulatory function in mammalian muscle growth. Further supporting this idea, phylogenetic analyses of nine mammalian species revealed that skeletal muscle exhibits the lowest evolutionary divergence in transcriptional output and resilience compared with organs such as lung, adipose tissue, kidney, spleen, heart, and liver [27]. This finding confirms that skeletal muscle is a primary target of ZBED6 and suggests that the ZBED6-mediated regulatory network is highly conserved among mammals. Based on these findings, we believe that the ZBED6-FNDC1 regulatory pathway revealed in this study has important cross-species applications. First, this pathway can be used as a molecular framework for analyzing the common patterns of mammalian muscle growth, providing a new entry point for comparative biology. Secondly, in the field of breeding, the *ZBED6* gene editing strategy based on the porcine model may be applicable to the improvement of muscle traits in other economically important animals, such as cattle and sheep, thereby providing a new molecular breeding target for improving meat production efficiency. Therefore, the present study not only deepened the understanding of the functional mechanism of ZBED6, but also laid an important theoretical foundation for cross-species muscle growth regulation.

Previous studies have shown that ZBED6 is able to regulate the Ras signaling pathway through IGF2, which in turn affects skeletal muscle growth in mice [13]. Notably, we previously showed that, in the ZBED6-deficient pig model of sepsis, *DOCK3* expression was upregulated in skeletal muscle and myocytes, activating the RAC1/PI3K/AKT pathway, which helped to alleviate sepsis-induced muscle atrophy [15]. In previous studies, our group also demonstrated that FNDC1 directly promotes mouse myogenesis through activating the FAK/PI3K/AKT/mTOR signaling pathway [16]. Based on this evidence, the present study showed that *ZBED6* deficiency could also activate FNDC1-mediated PI3K/AKT and MAPK/ERK1/2 signaling pathways and promote porcine myogenesis. Taken together, our findings support a model in which ZBED6 contributes to primary porcine skeletal muscle satellite cell proliferation and differentiation through multiple downstream targets, including *FNDC1* and *IGF2*. Among these, *FNDC1* represents an important contributor to PI3K/AKT and MAPK/ERK1/2 signaling activation, thereby complementing the previously established role of *IGF2* in ZBED6-mediated regulation of muscle growth.

## Conclusions

In this study, we revealed the important role of the *ZBED6-FNDC1* axis in myogenesis and verified its regulatory mechanism through cellular experiments. However, the study was mainly based on a primary porcine skeletal muscle satellite cell model, which, although providing important clues for understanding the mechanism, may not accurately reflect the complex physiological environment in vivo or fully capture the regulatory role of the *ZBED6-FNDC1* axis in the whole organism. Therefore, future studies are needed to elucidate these questions directly in pigs.

## Supplementary Information

Additional file 1: Fig. S1. Validation of *ZBED6* and *FNDC1 *knockdown efficiency in the primary porcine skeletal muscle satellite cells. Fig. S2. *FNDC1* promotes proliferation of ZBED6-deficient primary porcine skeletal muscle satellite cells independently of *IGF2*. Fig. S3. Validation of *ZBED6* and *FNDC1* overexpression efficiency in the primary porcine skeletal muscle satellite cells. Fig. S4. Effects of *FNDC1* or *IGF2* knockdown on myogenic differentiation in ZBED6-deficient primary porcine skeletal muscle satellite cells. Fig. S5. Integrative pathway analyses identify PI3K/AKT and MAPK signaling as common downstream pathways regulated by the *ZBED6-FNDC1* axis.

Additional file 2: The original gel and blot images.

## Data Availability

Publicly available in a repository: RNA sequencing was performed in previous study. Raw data for downstream signaling pathway analyses have been submitted to NCBI Gene Expression Omnibus (http://www.ncbi.nlm.nih.gov/geo/) under accession number GSE217125, PRJNA663759.

## Abbreviations

AKT: Protein kinase B (PKB)
CCND1: Cyclin D1
CCNE1: Cyclin E1
CDK4: Cyclin-dependent kinase 4
CDKN1A: Cyclin-dependent kinase inhibitor 1A
cDNA: Complementary DNA
CXCL12: C-X-C motif chemokine ligand 12
DOCK3: Dedicator of cytokinesis 3
EGR2: Early growth response 2
ERK: Extracellular signal-regulated kinase
FNDC1: Fibronectin type III domain containing 1
GAPDH: Glyceraldehyde-3-phosphate dehydrogenase
IGF2: Insulin-like growth factor 2
JNK: C-Jun N-terminal kinase
mTOR: Mechanistic target of rapamycin
MYF5: Myogenic factor 5
MYHC: Myosin heavy chain
MYOD: Myogenic differentiation 1
MYOG: Myogenin
PCNA: Proliferating cell nuclear antigen
p38: P38 mitogen-activated protein kinase (p38 MAPK)
QTL: Quantitative trait loci
qPCR: Real-time quantitative polymerase chain reaction
SCs: Satellite cells
SOBP: Sine oculis binding protein homolog
ZBED6: Zinc finger BED-type containing 6
