# Supplementary material for: ZBED6 deletion activates FNDC1-mediated PI3K/AKT and MAPK/ERK1/2 signaling pathways to promote myogenesis in pigs
Source: J Anim Sci Biotechnol. 2026 Oct 5;17:187. doi: 10.1186/s40104-026-01504-w (PMC13637308; doi:10.1186/s40104-026-01504-w)
Supplement: Supplementary file 1 [file 40104_2026_1504_MOESM1_ESM.docx]

**Fig. S1. Validation of *ZBED6* and *FNDC1* knockdown efficiency in the primary porcine skeletal muscle satellite cells.** **A.** Relative *ZBED6* mRNA expression following transfection with si-*ZBED6* or negative control (si-NC) in pig primary muscle satellite cells, *n* = 6 independent cell-culture experiments. **B.** Relative *FNDC1* mRNA expression following transfection with si-*FNDC1* or si-NC in pig primary muscle satellite cells, *n* = 6 independent cell-culture experiments.

**Fig. S2.** ***FNDC1* promotes proliferation of ZBED6-deficient porcine skeletal muscle satellite cells independently of *IGF2*. A.** Effect of *IGF2* knockdown on *FNDC1* mRNA expression in ZBED6-deficient pig primary muscle satellite cells. **B.** Relative *IGF2* mRNA expression levels in ZBED6-deficient primary porcine muscle satellite cells following *FNDC1* knockdown, *n* = 6 independent cell-culture experiments. **C and D.** Representative immunofluorescence for EdU and quantification of EdU-positive cells in ZBED6-deficient cells transfected with si-*FNDC1* or si-*IGF2*, stained for EdU (green) and nuclei (blue), *n* = 3 independent cell-culture experiments. Scale bar: 150 μm.

**Fig. S3 Validation of *ZBED6* and *FNDC1* overexpression efficiency in the primary porcine skeletal muscle satellite cells.**

**A.** Relative *ZBED6* mRNA expression following transfection with the *ZBED6* overexpression plasmid (*ZBED6*-OE) or empty vector (pcDNA3.1) in pig primary muscle satellite cells, *n* = 6 independent cell-culture experiments. **B.** Relative *FNDC1* mRNA expression following transfection with the *FNDC1* overexpression plasmid (*FNDC1*-OE) or pcDNA3.1 in pig primary muscle satellite cells, *n* = 6 independent cell-culture experiments.

**Fig. S4.** **Effects of *FNDC1* or *IGF2* knockdown on myogenic differentiation in ZBED6-deficient porcine skeletal muscle satellite cells.**

**A and B.** Representative immunofluorescence for MYHC and fusion index in ZBED6-deficient cells transfected with si-*FNDC1* or si-*IGF2*, stained for MYHC (red) and nuclei (blue), *n* = 3 independent cell-culture experiments. Scale bar: 300 μm.

**Fig. S5. Integrative pathway analyses identify PI3K/AKT and MAPK signaling as common downstream pathways regulated by the ZBED6–FNDC1 axis.**

**A and B.** Gene set enrichment analysis (GSEA) of the PI3K–AKT and MAPK signaling pathways in skeletal muscle of ZBED6-deficient pigs. **C and D.** Gene set enrichment analysis (GSEA) of the PI3K–AKT and MAPK signaling pathways in *FNDC1* knockdown C2C12 cells. **E.** Signaling pathway analysis of differentially expressed genes in myoblast from *ZBED6* knockout C2C12 cells.
